# 6-(Methylsulfinyl) Hexyl Isothiocyanate Inhibits IL-6 and CXCL10 Production in TNF-α-Stimulated Human Oral Epithelial Cells

**DOI:** 10.3390/cimb44070201

**Published:** 2022-06-29

**Authors:** Masahiro Shimoyama, Yoshitaka Hosokawa, Ikuko Hosokawa, Kazumi Ozaki, Keiichi Hosaka

**Affiliations:** 1 Department of Regenerative Dental Medicine, Institute of Biomedical Sciences, Tokushima University Graduate School, Tokushima 770-8504, Japan; c301751013@tokushima-u.ac.jp (M.S.); ihosokawa@tokushima-u.ac.jp (I.H.); hosaka@tokushima-u.ac.jp (K.H.); 2 Department of Oral Health Care Promotion, Institute of Biomedical Sciences, Tokushima University Graduate School, Tokushima 770-8504, Japan; ozaki@tokushima-u.ac.jp

**Keywords:** 6-MSITC, anti-inflammatory effect, oral epithelial cells

## Abstract

6-(Methylsulfinyl) hexyl isothiocyanate (6-MSITC) is a bioactive substance found in wasabi (Wasabia japonica) and has been reported to have some bioactive effects including anticancer and antioxidant effects. However, there are no reports on its effects on periodontal resident cells, and many points remain unclear. In this study, we aimed to investigate whether 6-MSITC exerts anti-inflammatory effects on human oral epithelial cells, including effects on signal transduction pathway activation. 6-MSITC inhibited interleukin (IL)-6 and C-X-C motif chemokine ligand 10 (CXCL10) production in TNF-α-stimulated TR146 cells, which are a human oral epithelial cell line. Moreover, we found that 6-MSITC could suppress signal transducer and activator of transcription (STAT)3, nuclear factor (NF)-κB, and p70S6 kinase (p70S6K)-S6 ribosomal protein (S6) pathways activation in TNF-α-stimulated TR146 cells. Furthermore, STAT3 and NF-κB inhibitors could suppress IL-6 and CXCL10 production in TNF-α-treated TR146 cells. In summary, 6-MSITC could decrease IL-6 and CXCL10 production in human oral epithelial cell by inhibiting STAT3 and NF-κB activation.

## 1. Introduction

Periodontitis is an inflammatory disease induced by bacteria [1]. An excessive immune response to bacteria is responsible for periodontal tissue destruction [2]. Currently, antibiotics administered locally to periodontal lesions are used for treatment, but the problem of resistant bacteria has been pointed out [3], and new anti-inflammatory substances are expected to be discovered.

6-(Methylsulfinyl) hexyl isothiocyanate (6-MSITC) is a substance found in wasabi, which is a popular spice used in Japanese cuisine such as sushi. Recently, several bioactive actions have been reported. Yano et al. reported that 6-MSITC inhibited the cell proliferation and induced apoptosis in human colorectal cancer cells via *p53*-independent mitochondrial dysfunction pathway [4]. Fuke et al. also reported that 6-MSITC promotes apoptosis of breast cancer cells by inhibiting nuclear factor (NF)-κB pathway [5]. However, there have been no reports examining the bioactive effects of 6-MSITC on periodontal tissue component cells, and there have been no attempts to use 6-MSITC for periodontitis treatment.

This study focused on the anti-inflammatory effects of 6-MSITC on human oral epithelial cells. Namely, we analyzed the effects of 6-MSITC on the production of interleukin (IL)-6, which is involved in osteoclast differentiation, and C-X-C motif chemokine ligand 10 (CXCL10), which is involved in Th1 cell infiltration. The effects of 6-MSITC on the activation of signaling pathways (signal transducer and activator of transcription 3 (STAT3), NF-κB and p70S6 kinase (p70S6K)-S6 ribosomal protein (S6)) were also investigated.

## 2. Materials and Methods

### 2.1. Cell Culture

Dr. Mark Herzberg provided TR146 cell line which is a human oral epithelial cell line. We used TR146 cells in this study. TR146 cells were cultured in Ham’s F12 medium (Nakarai Tesque, Kyoto, Japan) containing 10% fetal bovine serum (FBS) (JRH Biosciences, Lenexa, KS, USA), 1 mmol/L sodium pyruvate (Gibco, Grand Island, MI, USA), and antibiotics (penicillin G, 100 units/mL; streptomycin, 100 μg/mL; Gibco) at 37 °C in a humidified atmosphere with 5% CO_2_. When the cells reached subconfluency, the cells were harvested for subculture using a 0.25% trypsin-EDTA solution.

### 2.2. Cytotoxicity Assay

Cell viability was determined using Cell Count Reagent SF (Nakarai Tesque). Briefly, TR146 cells were seeded in 96-well plates and incubated for 2 days. After 2 days, the media was removed and 90 μL of Ham’s F12 medium at different concentrations of 6-MSITC (Cayman Chemical, Ann Arbor, MI, USA) were added and the cells were incubated for another 24 h. Then, we added 10 μL Cell Count Reagent SF and incubated the cells for 2 h, and measured the absorbance at 450 nm by microplate reader. 

### 2.3. IL-6 and CXCL10 Production in TR146 Cells

Sub-confluent of TR146 cells were stimulated with TNF-α (Peprotech, Rocky Hill, NJ, USA) with or without 6-MSITC, LY2584702 (a p70S6K inhibitor: Cayman Chemical), 5, 15-DPP (a STAT3 inhibitor: Cayman Chemical), SC514 (a NF-κB inhibitor: Cayman Chemical) for 24 h. The concentration of IL-6 and CXCL10 in the cell culture supernatant of TR146 cells was determined by DuoSet ELISA Development Systems (R&D systems, Minneapolis, MN, USA) in accordance with the manufacturer’s instructions. 

### 2.4. Western Blot Analysis

TR146 cells were cultured in 12-well plates and collected in cell lysis buffer (Cell Signaling Technology, Danvers, MA, USA) after TNF-α (100 ng/mL) stimulation for 15, 30, or 60 min with or without 6-MSITC (25 μM) pretreatment for 1 h. The protein concentrations in the lysates were measured by BCA Protein Assay Kit (TaKaRa, Shiga, Japan). An equal amount of protein was loaded onto a 4–20% SDS-PAGE gel followed by electrotransfer to a polyvinylidene difluoride membrane. Firstly, the membrane was incubated with phospho-STAT3 rabbit monoclonal antibody (Cell Signaling Technology), STAT3 mouse monoclonal antibody (Cell Signaling Technology), phospho-NF-κB p65 rabbit monoclonal antibody (Cell Signaling Technology), NF-κB p65 rabbit monoclonal antibody (Cell Signaling Technology), phospho-IκB-α mouse monoclonal antibody (Cell Signaling Technology), IκB-α mouse monoclonal antibody (Cell Signaling Technology), phospho-p70S6K rabbit monoclonal antibody (Cell Signaling Technology), p70S6K rabbit monoclonal antibody (Cell Signaling Technology), phospho-S6 rabbit monoclonal antibody (Cell Signaling Technology), S6 rabbit monoclonal antibody (Cell Signaling Technology), or Glyceraldehyde-3-phosphate dehydrogenase (GAPDH) rabbit monoclonal antibody (Cell Signaling Technology). After washing the membrane, the membrane was reacted with horseradish peroxidase-conjugated secondary antibody (Sigma-Aldrich, St. Louis, MO, USA). Protein bands were visualized on X-ray films by the ECL Prime Western Blotting Detection system (Cytiva, Tokyo, Japan). 

### 2.5. Statistical Analysis

Statistical significance was analyzed using one-way ANOVA followed by a post-hoc Tukey–Kramer test. *p* values of less than 0.05 were considered significant.

## 3. Results

### 3.1. Effects of 6-MSITC on Cell Viability of TR146 Cells

**Figure 1:**
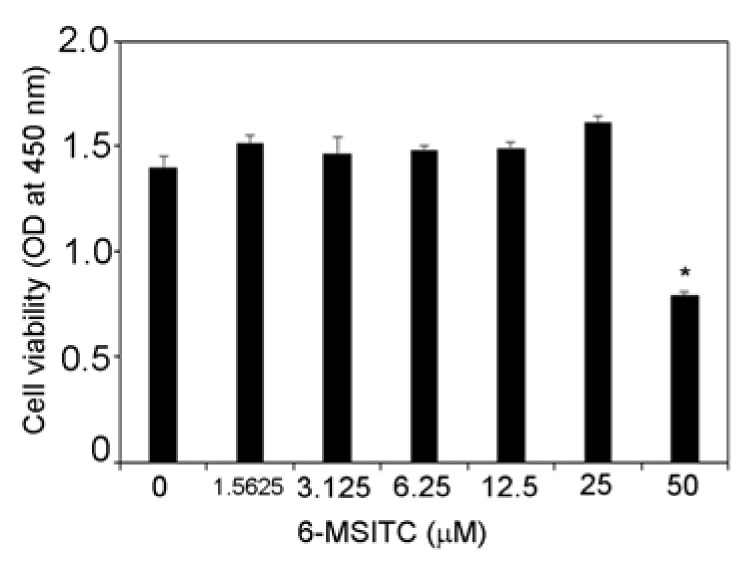
**Effects of 6-MSITC on viability of TR146 cells.** TR146 cells were seeded on 96-well cell culture plates, cultured for 2 days, and then treated with 6-MSITC (1.5625–50 μM) for 24 h. Cell viability was assessed using Cell Count Reagent SF. Data are expressed as the mean ± SD of 4 independent experiments. * = *p* < 0.05, significantly different from the TR146 cells without 6-MSITC.

At first, we examined the effect of 6-MSITC on the viability of TR146 cells. Figure 1 shows that 6-MSITC (1.5625–25 μM) did not influence the viability of TR146 cells. Therefore, 6-MSITC (1.5625–25 μM) was used for this study.

### 3.2. 6-MSITC Inhibits TNF-α-Induced IL-6 and CXCL10 Production in TR146 Cells

**Figure 2:**
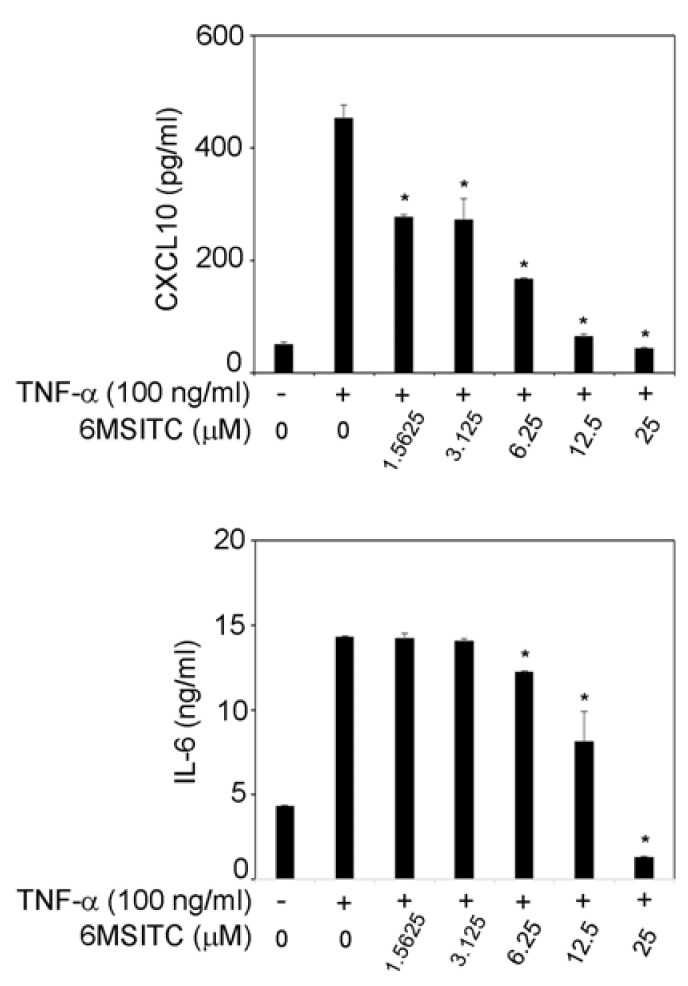
**Effects of 6-MSITC on TNF-α-induced production of CXCL10 and IL-6.** Sub-confluent TR146 cells were cultured with TNF-α (100 ng/mL) with or without 6-MSITC (1.5625–25 μM) for 24 h. The amounts of CXCL10 and IL-6 in the supernatant were determined using their respective ELISA kits as described in the Materials and Methods section. Data are expressed as the mean ± SD of 3 independent experiments. * = *p* < 0.05, significantly different from the TR146 cells stimulated with TNF-α without 6-MSITC.

Figure 2 shows that TNF-α (100 ng/mL) increased IL-6 and CXCL10 release in TR146 cells. When 1.5625–25 μM 6-MSITC was added, CXCL10 production in TNF-α-stimulated TR146 cells was significantly inhibited (Figure 2). TNF-α-induced IL-6 production in TR146 cells was decreased by 6.25–25 μM 6-MSITC treatment (Figure 2).

### 3.3. Effects of STAT3, NF-κB, and p70S6K-S6 Signaling Pathways in TR146 Cells

**Figure 3:**
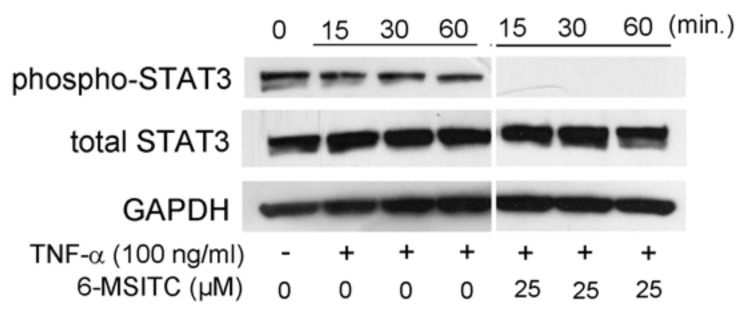
**6-MSITC mediated inhibition of a STAT3 pathway in TNF-α stimulated TR146 cells.** After pretreatment with 6-MSITC (25 μM) for one hour, TR146 cells were stimulated with TNF-α for 15, 30, or 60 min, and then phosphorylation of STAT3 was determined by Western immunoblotting. The figure reports the results of a representative experiment performed three times with qualitatively comparable results.

**Figure 4:**
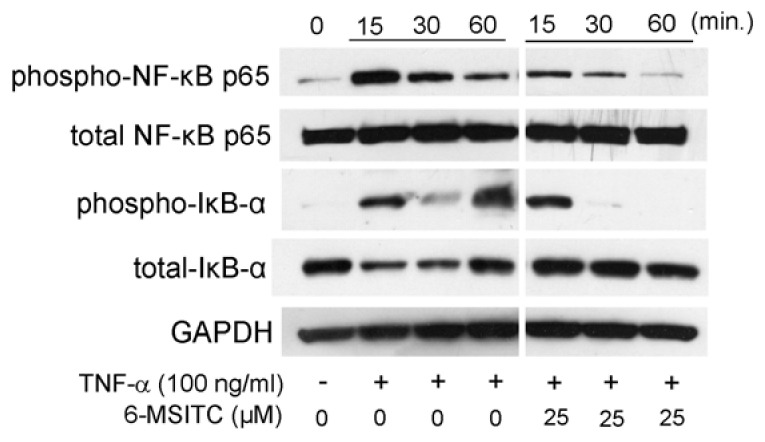
**6-MSITC mediated inhibition of a NF-κB pathway in TNF-α stimulated TR146 cells.** After pretreatment with 6-MSITC (25 μM) for one hour, TR146 cells were stimulated with TNF-α for 15, 30, or 60 min, and then phosphorylation of NF-κB p65 and IκB-α, and IκB-α degradation were determined by Western immunoblotting. The figure reports the results of a representative experiment performed three times with qualitatively comparable results.

**Figure 5:**
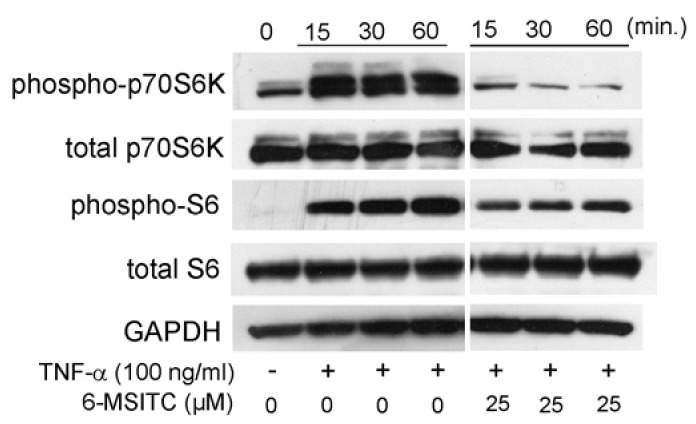
**6-MSITC mediated inhibition of a p70S6K-S6 pathway in TNF-α stimulated TR146 cells.** After pretreatment with 6-MSITC (25 μM) for one hour, TR146 cells were stimulated with TNF-α for 15, 30, or 60 min, and then phosphorylation of p70S6K and S6 was determined by Western immunoblotting. The figure reports the results of a representative experiment performed three times with qualitatively comparable results.

It is certain that STAT3, NF-κB, and p70S6K-S6 signaling pathways are activated in TNF-α-stimulated TR146 cells. Therefore, we would like to know the effects of 6-MSITC on the activation of the aforementioned signaling pathways. Figure 3 shows that 25 μM 6-MSITC completely inhibited TNF-α-induced the phosphorylation of STAT3 in TR146 cells. Figure 4 shows that the level of NF-κB p65 phosphorylation was decreased by 6-MSITC treatment, and IκB-α phosphorylation was clearly inhibited by 6-MSITC pretreatment at 60 min. IκB-α degradation was also inhibited by 6-MSITC treatment. The levels of p70S6K and S6 phosphorylation in TNF-α-stimulated TR146 cells were also clearly decreased by 6-MSITC treatment (Figure 5). These data show that 6-MSITC pretreatment could inhibit multiple signaling pathways including STAT3, NF-κB, and p70S6K-S6 at the same time in TR146 cells.

### 3.4. STAT3 and NF-κB Signaling Pathways Are Involved in IL-6 and CXCL10 Production in TNF-α-Treated TR146 Cells

**Figure 6:**
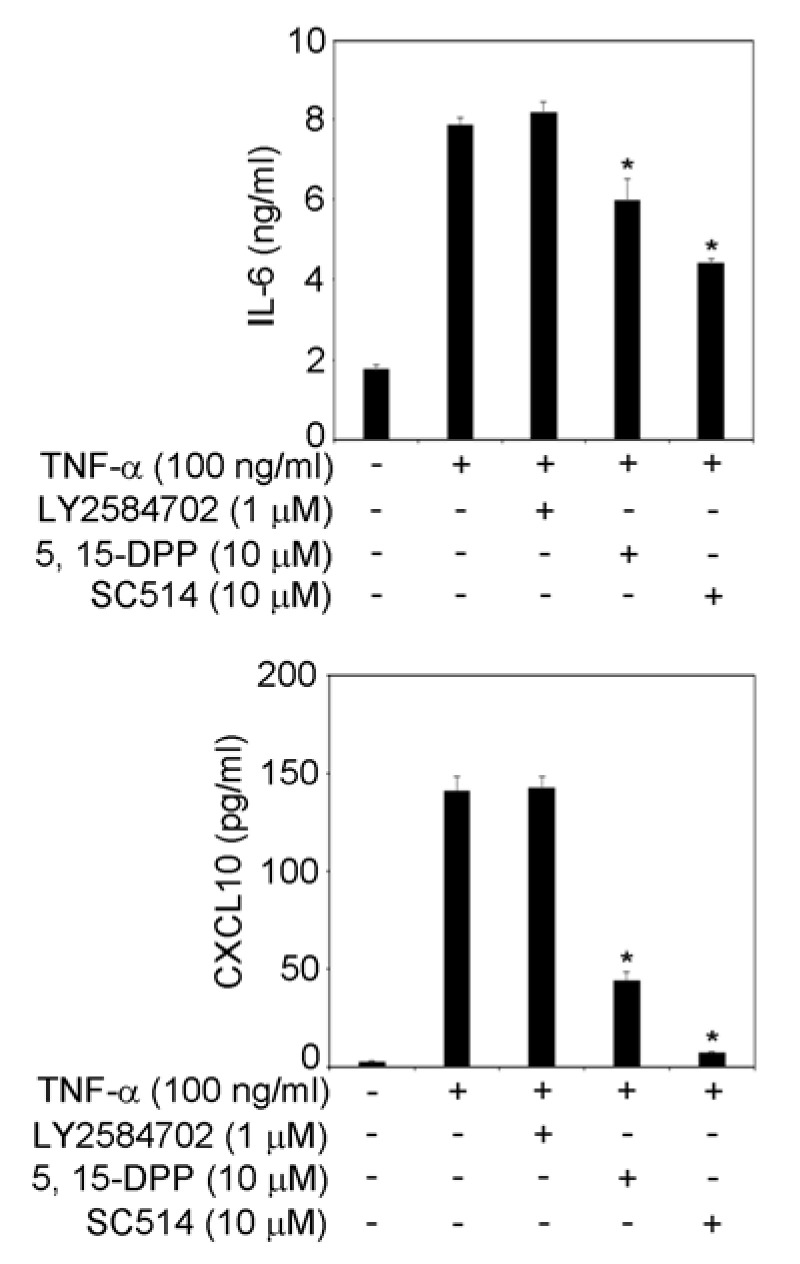
**Effects of signal transduction inhibitors on TNF-α-induced production of CXCL10 and IL-6.** Sub-confluent TR146 cells were cultured with TNF-α (100 ng/mL) with or without LY2584702 (I μM), 5, 15-DPP (10 μM), or SC514 (10 μM) for 24 h. The amounts of CXCL10 and IL-6 in the supernatant were determined using their respective ELISA kits as described in the Materials and Methods section. Data are expressed as the mean ± SD of 3 independent experiments. * = *p* < 0.05, significantly different from the TR146 cells stimulated with TNF-α without signal transduction inhibitors.

Figure 3, Figure 4 and Figure 5 show that 6-MSITC could inhibit the activation of STAT3, NF-κB, and p70S6K-S6 signaling pathways. Finally, we examined which signaling pathways control IL-6 and CXCL10 production in TR146 cells by signal transduction inhibitors. Figure 6 shows that a STAT3 inhibitor and a NF-κB inhibitor significantly decreased TNF-α-induced IL-6 and CXCL10 production in TR146 cells. This result may suggest that the attenuation of TNF-α-induced IL-6 and CXCL10 production in TR146 cells by 6-MSITC is due to STAT3 and NF-κB signaling pathways inhibition.

## 4. Discussion

Periodontitis is a chronic inflammatory disease induced by periodontopathogenic bacteria. Local administration of antimicrobial agents has been used in the treatment of periodontitis, but recently, due to the problem of bacterial resistance, the discovery of new bioactive substances with anti-inflammatory activity has been desired. We focused on bioactive substances contained in wasabi, which is commonly used in Japan. This is because we believe that Japanese people can use it safely.

In this study, we showed 6-MSITC could inhibit IL-6 and CXCL10 production in TNF-α-stimulated TR146 cells. There have been several papers mentioning the anti-inflammatory effects of 6-MSITC. Chen et al. reported that microarray analysis showed 6-MSITC could suppress several inflammatory mediator mRNA expressions in lipopolysaccharide (LPS)-stimulated murine macrophage-like RAW264 cells [6]. Okamoto et al. also reported that 6-MSITC decreased IL-6 and CC chemokine ligand (CCL)2 production in TNF-α-stimulated human umbilical vein endothelial cells [7]. Judging from previous reports and ours, 6-MSITC is likely to have anti-inflammatory effects on a variety of cells. Further studies are necessary because there are currently few reports on the anti-inflammatory effects of 6-MSITC.

NF-κB is a major signaling pathway involved in inflammatory mediator production and has been reported to be involved in IL-6 [8] and CXCL10 [9] production. In this study, we showed that 6-MSITC inhibits NF-κB p65 and IκB-α phosphorylation in TR146 cells. It has been reported that 6-MSITC inhibits the NF-κB pathway by suppressing the phosphorylation of IκB-α in human breast cancer cells [5]. The NF-κB pathway was inhibited in both normal and cancer cells, indicating that suppression of the NF-κB pathway activation may be the main bioactive action of 6-MSITC.

It has been reported that TNF-α stimulation activates the STAT3 signaling pathway [10]. Therefore, we examined whether 6-MSITC inhibits STAT3 phosphorylation. Our study showed that 6-MSITC can clearly inhibit STAT3 activation. No previous studies have examined the effect of 6-MSITC on STAT3 activation. However, there have been reports of the effects of other isothiocyanates on STAT3. For example, sulforaphane, a well-known isothiocyanate, has been reported to suppress STAT3 activation in a hepatocellular carcinoma cell line [11]. Hence, inhibition of the STAT3 signaling pathway may be a common feature of isothiocyanates. Further studies are necessary to prove the hypothesis.

It is certain that sulforaphane inhibits the p70S6K-S6 pathway in several types of cells [12,13]. Therefore, we examined whether 6-MSITC could modulate the p70S6K-S6 pathway in TNF-α-stimulated TR146 cells. Figure 5 clearly shows that 6-MSITC treatment decreased the level of p70S6K and S6 phosphorylation in TR146 cells. However, a p70S6K inhibitor did not inhibit IL-6 and CXCL10 production. This fact indicates that inhibition of the p70S6K-S6 pathway by 6-MSITC is not associated with decreased IL-6 and CXCL10 production. It is generally known that phosphorylation of S6 increases protein synthesis and cell proliferation [14]. Our next task is to elucidate the effect of S6 phosphorylation on human oral epithelial cells.

Uto et al. previously reported that 6-MSITC could inhibit p38 mitogen-activated protein kinase (MAPK), extracellular signal-regulated kinase (ERK), and c-Jun N-terminal kinase (JNK) phosphorylation in LPS-stimulated mouse macrophages [15]. It is certain that TNF-α stimulation could activate MAPKs pathways in various kinds of cells. Therefore, we should examine the effects of 6-MSITC on MAPKs pathways in TNF-α-stimulated human oral epithelial cells in our next studies. 

It is unknown how cells recognize 6-MSITC. However, sulforaphane, an isothiocyanate, has recently been reported to bind to P2Y6 receptors [16], and it is possible that 6-MSITC may bind to cells by the same mechanism. We think further studies are needed to prove the hypothesis.

In summary, 6-MSITC could suppress IL-6 and CXCL10 production in TNF-α-treated human oral epithelial cells (TR146 cells) by inhibiting the activation of STAT3 and NF-κB pathways. Thus, the administration of 6-MSITC to the periodontal lesion may be considered in treatment of periodontitis. Further studies using other periodontal tissue component cells or animal periodontitis models are warranted.

## Data Availability

Not applicable.
